# The Development of Parental Knowledge of Their Child in NNU and PICU: An Exploratory Qualitative Study of Parents' Experiences

**DOI:** 10.1111/nicc.70593

**Published:** 2026-07-27

**Authors:** Sarah E. Seaton, Ashleigh E. Butler, Joseph C. Manning, Lucy K. Smith, Katie Gallagher, Nicola Mackintosh

**Affiliations:** ^1^ School of Healthcare University of Leicester Leicester UK; ^2^ School of Nursing and Midwifery La Trobe University Melbourne Australia; ^3^ Nottingham Children's Hospital Nottingham University Hospitals NHS Trust Nottingham UK; ^4^ Neonatal Service University Hospitals of Leicester NHS Trust Leicester UK; ^5^ School of Medical Sciences University of Leicester Leicester UK

**Keywords:** neonatal care, paediatric intensive care, parent experience, qualitative research

## Abstract

**Background:**

Advancements in neonatal and paediatric intensive care have improved survival of children born very preterm or with complex health conditions. However, many of these children will subsequently require admission to paediatric intensive care. How families navigate experiences and knowledge across these different admissions remains poorly understood.

**Aim:**

To explore the experiences of parents with a child admitted to both a neonatal unit (NNU) and a paediatric intensive care unit (PICU) with a focus on the development of parental knowledge.

**Study Design:**

We conducted an exploratory qualitative study. Semi‐structured interviews were conducted with 18 parents of 15 children who experienced admission to both NNU and PICU. Participants were recruited via national charities and interviewed remotely. Data were analysed using inductive content analysis, focussing on parents' temporal experiences.

**Findings:**

Parents described knowing their child as a dynamic state evolving throughout the NNU to the PICU. In the NNU, parents initially learned to know their child through hands‐on care, often facilitated by staff, though physical and emotional barriers sometimes hindered bonding. Discharge from NNU was a key transition moment, with some parents feeling confident while others felt uncertain about managing their child's healthcare needs at home without the support of staff and medical equipment. At PICU admission, parents brought experiential knowledge, the value of which was not always recognised by healthcare professionals. This lack of acknowledgement sometimes led to missed opportunities for provision of safe care. During PICU stays, parents wanted their expertise respected and integrated into care, particularly for children requiring ongoing specialist care, which was often provided by families, outside of PICU.

**Conclusions:**

Parental knowledge of their child, and their previous experiences, is vital to support safe care delivery but sometimes overlooked. Recognising parents as expert and equal partners in care can potentially improve safety, communication and family‐centred practice.

**Relevance for Clinical Practice:**

Neonatal and paediatric healthcare teams should actively acknowledge and incorporate parental expertise during and when transitioning between NNU and PICU. Parents' experiences provide additional insights, which provide opportunities for better care. Improved communication across neonatal and paediatric services may enhance outcomes and parental confidence.

## Impact Statement

- What is known about this topic
  ○ Advancements in neonatal and paediatric care have significantly improved survival and outcomes, meaning more children are spending time in both settings.
  ○ Transitions between healthcare services, including neonatal and paediatric, are known to be moments that can disrupt continuity of care.
- What this paper adds
  ○ Parental knowledge of their child develops dynamically across neonatal and paediatric settings and experiences.
  ○ There are more opportunities to involve parents who often hold expert, experience‐based knowledge that is not always recognised or incorporated into care.

## 1 Introduction

### 1.1 Background

Advancements in neonatal and paediatric intensive care have significantly improved survival rates for babies born very preterm or with complex medical conditions [1, 2]. These conditions are varied but may include respiratory problems associated with preterm birth or complications arising from congenital anomalies. Subsequently, there are increasing numbers of children living with chronic and lifelong health conditions that often have their origins in the antenatal and neonatal period. This group of children forms a subset of those often described as living with ‘medical complexity’ or ‘chronic critical illness’ but as yet there is no clear consensus on what medical conditions meet these criteria. While survival has improved, the long‐term health and well‐being needs of these children are less clear, but potentially place greater demands on families and healthcare systems.

In both neonatal units (NNU) and paediatric intensive care units (PICU), there are efforts to facilitate bonding between the child and their family to mitigate the separation resulting from admission, through philosophies of family‐integrated care [3] and family‐centred care [4]. Developing a relationship with and knowledge of their child is essential for parents, particularly in the context of critical care environments or living with lifelong conditions. This development process enables them to be better equipped to take on the role of being advocates for their child. Supporting parents in building and trusting this knowledge is therefore a key component of family‐centred care.

While qualitative research has explored the emotional, psychological and practical experiences of families whose children are admitted to either neonatal care or paediatric intensive care [5], there is less research about families who navigate both experiences. The experiences of admission to both neonatal and paediatric environments provide an additional level of complexity for these families, who may face prolonged and repeated interactions with critical care services in different settings leading to periods of separation, impacting on the ability to form meaningful bonds as a family. Little is known about the journeys of these families and whether aspects of a prior NNU admission support parental role expectations in subsequent PICU admissions.

### 1.2 Aim and Objectives

To explore the experiences of parents with a child admitted to both a neonatal unit (NNU) and a paediatric intensive care unit (PICU) with a focus on the development of parental knowledge.

## 2 Design and Methods

### 2.1 Setting and Sample

We recruited a national, purposive sample of parents with experiences of having a child cared for in a NNU (after birth) and PICU (before age of three), with the most recent PICU interaction having been within the last 10 years. We subscribed to a constructivist epistemology, where knowledge is generated between the researcher and the participants, and there is no single reality of the world, with all experiences equally valid and real.

In this work when we refer to parents, we mean this in the broadest sense to encompass anyone with a caregiving role in a child's life. This includes, but is not limited to, biological/adoptive parents, step‐parents, grandparents and other family members, chosen or biological.

Study adverts were shared by charities that support families of sick children, who could also provide support to parents if needed, and on social media. Parents contacted the study team to participate and were provided with information leaflets and consent forms for completion in advance, with verbal reaffirmation of consent taken at the start of the interview. Sources of support were provided and an additional signposting leaflet to further support (e.g., charities) was available on request before, during or after the interview.

### 2.2 Data Collection Tools and Methods

Our study used a qualitative exploratory approach with semi‐structured interviews with parents of children admitted to NNU and PICU. A topic guide was developed from the literature and with input from parents with lived experience (summary in Appendix 1) and piloted on two parents. Interviews were held over Microsoft Teams or the telephone and recorded and transcribed by SES, with regular debriefs with NM.

### 2.3 Reflexivity and Rigour

The lead researcher (SES) who completed all interviews and coding is a cisgender child‐free woman with several years of experience in paediatric epidemiology. SES had no prior relationship with the participants; however, her professional background may shape her perspectives of safety, transitions and family involvement. The other authors are an interdisciplinary team of researchers, social scientists and nurses with expertise in both neonatal and paediatric critical care. Therefore, to enhance rigour and trustworthiness, we carefully considered quality criteria of credibility; transferability; dependability; and confirmability [6]. We held regular discussions as a research team to reflect on interpretations, challenge assumptions and enhance analytic rigour.

We were guided by meaning saturation and focussing on our overall research question we collected data until no new meaningful information was gained [7]. We adhered to the COnsolidated criteria for Reporting Qualitative research checklist [8].

### 2.4 Data Analysis

Inductive content analysis [9] was used to identify key themes of temporal parental experiences, with the aid of the NVivo 13 software. This analysis approach used a step‐by‐step process of: (1) reading the interviews; (2) identifying big‐picture meaning units; (3) developing subcategories; (4) refining the subcategories; and (5) synthesis and interpretation. In our work, this followed the process of all interviews were reviewed several times to develop familiarity with the data (Step 1). After immersion in the data, big‐picture units related to NNU experiences, PICU experiences, or NNU–PICU comparison experiences were identified, with relevant data related to each meaning unit extracted into tables (Step 2). Through open coding, the data extracts related to each of these meaning units were organised further into six subcategories, which were reviewed and refined through regular team discussion (Steps 3 and 4): communication; environment; memories/prior experience; involvement; knowing their child; and separation. For the purposes of this paper, we have focussed on the main subcategory of ‘knowing their child’, reviewing the original data to ensure all relevant experiences were captured (Step 5). Synthesis was undertaken by exploring similarities, differences and temporal elements between and within the codes and through team discussion and review, until a rich description of parental experiences of the NNU and PICU journey was developed. All steps of the analysis were discussed to maximise the trustworthiness of the findings.

### 2.5 Ethics Statement

Ethics approval was granted by the University of Leicester Medicine and Biological Sciences Research Ethics Committee (reference: 32037‐ses26‐ls:healthsciences) on 3rd November 2021.

## 3 Results/Findings

We interviewed 18 parents, 15 mothers and three fathers, of 15 children with experience of both NNU and PICU, with a variety of reasons for NNU (including antenatally diagnosed congenital anomalies, prematurity) and PICU (including recovery following cardiac surgery; respiratory illness) admissions (Table 1).

**TABLE 1:** Characteristics of parents interviewed and their child or children.^a^

Identifier	Age at the time of interview	Occupation	Sex of child	Information about the child or children who had been admitted to NNU and PICU
F001	31–40 years	Government worker	Boy	Born very preterm and admitted to PICU as an emergency with a respiratory condition.
F002	41–50 years	Healthcare professional	Girl	Born very preterm and admitted to PICU as an emergency with a respiratory condition. (Partner of M014) (interviewed separately).
F003	Not known	Not known	Boy	Born with a heart condition requiring planned PICU admission following surgery. Partner of M012 (interviewed separately).
M001	31–40 years	Healthcare professional	Girl	Born very preterm and admitted to PICU as an emergency with a respiratory condition.
M002	41–50 years	Civil engineer	Boy	Born with a heart condition requiring a planned PICU admission following surgery and a subsequent emergency admission for a neurological condition.
M003	Over 50 years	Government worker	Girl	Born with a heart condition requiring a planned PICU admission following surgery
M004	20–30 years	Carer for her child	Boy	Born with a genetic syndrome, which includes a heart condition requiring planned admission to PICU following surgery.
M005	31–40 years	Scientist	Girl	Born with a heart condition, which was not diagnosed antenatally and required a planned admission to PICU following surgery.
M006	31–40 years	N/K	Girl	Born very preterm and admitted to PICU as an emergency with a respiratory condition where she died aged 1 year.
M007	N/K	N/K	Boy	Respiratory difficulties after birth led to long‐term tracheostomy, which required planned PICU admission to be reversed.
M008	N/K	N/K	Girl	Born very preterm and admitted to PICU as an emergency with a respiratory condition.
M009	Over 50 years	N/K	Boy (one of triplets)	Born very preterm and planned admission to PICU with a neurological condition.
M010	41–50 years	Carer for her child	Boy	Born very preterm and with a cardiac condition. Planned transfers between PICU and neonatal care before continuing care in PICU.
M011	41–50 years	Healthcare professional	Boy	Born late preterm and experienced respiratory difficulties and admitted to PICU with suspected infection as an emergency.
M012	Not known	Not known	Boy	Born with a heart condition requiring planned PICU admission following surgery. Partner of F003 (interview separately).
M013	31–40 years	Healthcare professional	Boy and girl	Girl was born very preterm and admitted to the PICU as an emergency with a respiratory condition. Boy had an undetected cardiac condition and a planned admission to the PICU following surgery.
M014	41–50 years	Banker	Girl	Born very preterm and admitted to PICU with a respiratory condition as an emergency. (Partner of F002 (interviewed separately)).
M015	Not known	Not known	Boy	Born very preterm and planned transfer directly from neonatal care to PICU for ongoing respiratory support.

^a^ F denotes a father and M denotes a mother.

The concept of knowing their child was identified as underpinning parental experiences of NNU and PICU admissions. Primarily, the concept of knowing their child appeared when parents were talking about their experiences in PICU, or when comparing the NNU and PICU, but was also mentioned when recollecting NNU experiences. The state of knowing their child was not static and changed over time and at different stages for families. It was constantly evolving as the child's illness and care requirements changed across their journey through NNU, PICU and beyond.

### 3.1 Admission to NNU, Knowing Their Child: Learning to Know Their Child

Parents sometimes felt unprepared to meet their baby and even for those aware that their baby would require admission to the neonatal unit following an antenatal diagnosis, the transition to parenthood in the strange and unfamiliar environment of the NNU remained a challenge.

Some families relied on providing care for their child in the NNU as a potential way to bond with their child; however, this was not without challenge. While many parents recounted that NNU staff ‘were great at getting you involved, teaching you how to do cares, how to move her… (F002)’, the physical environment minimised opportunities to care for, and therefore get to know, their child: ‘s*he's in an incubator there's so little you can do. It was in a sense a real physical and emotional barrier between you and this baby (F002)’*, although they were encouraged to be ‘hands on (M003)’.

The uncertainty of their child's clinical course sometimes hindered their ability to bond with their child, and it was easier to focus on day‐to‐day practicalities rather than face the seriousness of the situation: ‘The first couple of days you just don't know what's going on, you sort of go with the flow a bit, in a little bit of denial cause obviously we didn't have a diagnosis. So, we were kind of hoping it was something minor and he'll be okay (M007)’.

For parents, confidence in their parenting role grew as they got to know their child in the NNU and became more involved in their care. For some parents whose baby had a prolonged stay, this could sometimes lead to conflict as they began to exert their parenting role: ‘…you don't want to come across as like telling them their job. But then equally I was the constant the whole way through (M010)’. Sometimes this led to disagreements about how to provide care: ‘she'd fallen asleep on me, like kangaroo care… and they were saying “no she'll sleep better in her cot, put her back down” and me saying “well actually I don't want to, she's settled” (M006)’. For parents of babies transferred between healthcare settings, seeing differences in care provision, and knowing what had previously helped their child, enabled them to feel confident to question the clinical teams: ‘because we got so involved at the [NNU1] and they encouraged that by the time we got to [NNU2]… We were very much going to the team “no we want to be as involved as we can be” (M006)’.

When parents reflected on time in the NNU, it was often remembered fondly as the place they had first got to know their child: ‘it was more friendly you were… involved in everything pretty much… they helped you to bond (M013)’. Many parents felt this initial experience of parenting and knowing their child in a critical care setting helped prepare them for events that happened later in their child's journey: *‘*I am glad I had that 3‐week NICU experience because I think they did help to nurture that in me and give me the strength almost say to when I went to PICU actually yeah I can do this (M010)’. This encouragement and support to bond with their child through hands‐on care and partnership with the neonatal team helped establish feelings of being confident parents without the safety net of the clinical environment.

### 3.2 Discharge From NNU, Knowing Their Child: Feeling Unsure of How to Care for Their Child

Discharge from the NNU marked a key transition point for families. Some families were happy, or desperate, to leave the NNU to begin or resume their independent life as a family: ‘I just became adamant that we were going home. I'd had enough and we were walking out of there (M001)’. These families often felt confident in their knowledge of their child and their care needs, and comfortable to take on this role at home: ‘I remember feeling desperate to just get home. You feel really confident that you're okay, you know what you are doing. She's gonna be okay (M003)’. The desire to be at home was sometimes compounded by other children (or other responsibilities) at home and wanting to have the experience of being a family together.

However, for some families, despite having got to know their child across the NNU journey, the prospect of discharge sometimes left parents feeling uncertain about how well they knew their child, and feeling anxious about coping at home without medical support. One father noted: ‘she was coming home with some complicated issues to consider and that was really terrifying. We didn't know, should we watch over her all night to make sure she's okay? (F002)’. These feelings were potentially compounded by the loss of practical support such as medical equipment and using their own skills to protect their child, with one mother noting: ‘no monitors and just ‘there we go, use your eyes to keep him alive!’ I found really hard (M011)’. Discharge from the NNU also meant that some families felt abandoned: ‘go and be a parent now in your own home (F001)’.

### 3.3 Navigating Admission to PICU, Knowing Their Child: Knowing Their Child's Needs

For some families, there was time between the NNU and PICU admission, with some families providing expert medical care at home unassisted. After readmission, some families had to advocate strongly for their child's condition to be taken seriously when they knew they were acutely unwell: ‘I'm saying “there something wrong with my baby” and I remember them going “yes mum, we hear you” kind of pat, pat on the back and walking away… and we were in surgery within an hour (M003)’ or to have their child's care stepped up to intensive care: ‘I was adamant that… her breathing was really not good… then ultimately PICU outreach came because I was getting really anxious… They were immediately like “what is going on here! Just get her on high flow” (M001)’.

Sometimes parents highlighted there were opportunities when they could have intervened because then their child may have benefited from a different course of treatment: ‘I should have stood up more in there and said don't do that, don't NG [nasogastric] feed him (M012)’. While the clinical details of these cases remain unknown, what was evident was that some parents held expert knowledge of their child which they felt was not respected or valued by the new healthcare team, leading to tension and missed opportunities to provide meaningful family‐centred care.

### 3.4 Time on PICU, Knowing Their Child: Knowing What Their Child Needs Best

All the families in this study had a subsequent admission to PICU, either directly after NNU or after a short period at home. Some had spent long periods in the NNU while others had had short stays. However, knowing their child was a temporally changing concept, and in the time since their child's birth, they had been able to get to know their child and process what had happened on the NNU: ‘we had processed what we been through with NICU. We could understand and deal with medical emergencies (F002)’, which significantly impacted their experiences and expectations of being a parent in the PICU. This was especially true for families who had spent longer at home between admissions. For these families, they had more time to know their children in a familiar context, which possibly made it harder to cope with leaving them in PICU, and having to re‐establish their parental role as an expert in their child's needs within the new context of the PICU: ‘we'd had like a whole year with her, we'd got to know her… in the PICU… you've been a parent for a year and then suddenly you've got to effectively hand your child over to somebody else. Which is very, very difficult to do (M008)’. For these families, admissions to PICU meant stepping back from their role as immediate caregiver and allowing someone else to take over this role.

Families often described themselves as having a skillset akin to a nurse and able to provide expert care to their child, and they struggled when PICU staff took the role from them: ‘staff need to take on board what the parent is already capable of doing because you know for a lot of PICU admissions they're quite complex children and actually the parents do almost the same job as a paediatric nurse on a daily basis at home. So don't suddenly take away from them all the things that are routine to what they do as they parent (M001)’.

While parents wanted the prior experiences of what they and their child had been through to be recognised, this could be easily overlooked, particularly if the child was still very young on admission to PICU: ‘this wasn't a baby that's been born at term and had got bronchiolitis at six weeks of age and was having an unexpected admission. There was a lot of baggage that was coming along and also a lot of experience (M001)’. While in NNU, they had been bonding and getting to know their child, and in PICU parents wanted to demonstrate they knew their child (and have this acknowledged) but this was sometimes hindered.

The medical knowledge of these families about their child's condition and care needs was substantial, but they also felt they knew when to ask for more information and to advocate for care to be properly explained when things were unfamiliar: ‘I knew what ECMO [extracorporeal membrane oxygenation] was because of another cardiac parent but my husband didn't know. So, I jumped straight in and I was like “I need you to explain more, like what's happening with him, why is that the case?” (M004)’. However, some families were disempowered from gaining all the information they wanted and had to negotiate access to knowledge: ‘They said “unless you're a microbiologist you don't need to know.” So, I think I obviously wanted to know too much information (M005)’.

Many families felt they had specialist knowledge of their child, which would have supported the delivery of care, such as how to distract their child during procedures, or knowledge of incidents during previous care, which were important for PICU staff to know. However, this knowledge was not always taken account of: ‘I said to the nurse that was on the night shift… he will pull ventilators out he will pull everything out that he can get his hands on and he's incredibly strong… So, she's like oh yes, don't worry dear it's fine, I know what I'm doing, and I came in the next morning and… he'd whipped it all out and he had to have the tube put back in (M010)’. In some circumstances, parents had information that could potentially prevent safety issues: ‘I was able to correct them and say “no that didn't happen” or “this happened” or “actually no he's this many weeks old” (M011)’ or were able to identify their child's potential to deteriorate: ‘I kept saying to them you know he'll go down really quickly and the nurses were saying “oh yes you know at the moment he's okay”… thank goodness this PICU consultant went “this child will surprise you” and then lo and behold within half an hour he was sinus [sinus tachycardia: fast heart rate] and sent down to PICU again (M009)’.

### 3.5 Discharge From PICU: Remembering Uncertainty in Knowing Their Child

Despite feeling like they knew their child and their needs on PICU discharge better than they did when first leaving the NNU, it was still an uncertain time for families. Some families found leaving PICU more manageable, particularly those that had some form of corrective surgery: ‘when you are sent home [from NNU] and told your baby is going to get sicker and sicker it's just awful. Whereas when we were sent home from [PICU] and you just know she's going to get stronger and stronger… and yeah, we were more knowledgeable by then (M005)’ or where PICU teams provided or facilitated additional knowledge for families (e.g., training in caring for tracheostomies). However, for other families, the subsequent admission to PICU had compromised their beliefs in how well they knew their child, realising how sick their child was or had been: ‘I think definitely my confidence in her being fine and just coping and us getting away from hospital was knocked significantly (M001)’. For some families, it affected their behaviour in terms of planning for the future, for example researching the locations of PICUs before taking journeys away from home.

For many families, while they worried about the future, when reflecting on the experiences in the NNU and PICU, they were more confident as parents after PICU: ‘We feel a lot more comfortable at managing any medical emergencies that arise so it felt very different, a very different experience. It felt really positive to come out of PICU whereas we didn't really have anything positivity coming home from NICU (F002)’.

## 4 Discussion

This study examined how parents develop and enact knowledge of their child across neonatal and paediatric intensive care settings. These environments are known to be distressing and overwhelming for families in the short [10] and longer term [11, 12] and may also hinder the natural bonding process of new parents with their child [13]. Our findings show that parental knowledge is dynamic, relational and shaped by transitions across care contexts. While these findings align with existing literature emphasising the importance of parental involvement, they also expose important tensions in how such involvement is enacted in practice.

### 4.1 Family‐Integrated Care

A key finding is the discrepancy between the policy ideal of family integrated care and its implementation in clinical settings. Family‐integrated care is often described as a partnership model in which parents and professionals share responsibility for care [3]. However, our findings suggest that parental involvement is actively negotiated rather than consistently embedded. Parents described both facilitation and constraint, indicating variability in how staff interpret and operationalise involvement. One explanation for this variation is the prioritisation of clinical stability and risk management within high‐acuity environments, which may lead professionals to limit parental participation. In contrast, parents positioned involvement as essential to bonding and the development of confidence in their caregiving role. These differing priorities highlight an underlying tension between biomedical knowledge and experiential, relational knowledge. It is known that significant health inequalities exist for babies and children from their earliest healthcare interactions [14, 15]. An unequitable approach to family‐integrated care implementation may compound this by creating a larger gap between families who are able to articulate and advocate for their baby. It is likely that our work did not fully appreciate these challenges as most families described themselves as White British, and everyone spoke English as a first language, and so we did not hear from the full range of families that are cared for in NNU and PICU.

### 4.2 Transition Points

This tension becomes more pronounced across transitions between NNU and PICU. Transitions are often understood as organisational challenges that disrupt continuity of care [16]. Our findings extend this by showing that transitions are also epistemic [17], as parental knowledge is re‐evaluated and sometimes devalued in new settings. Parents described arriving in PICU with substantial experiential knowledge yet being repositioned as inexperienced. This suggests that parental expertise is not fixed but is context‐dependent and contingent on recognition by healthcare professionals. Where this recognition is absent, opportunities for effective collaboration, and effective sharing of knowledge [18], may be reduced. Parents in our study often felt more confident in knowing their child after leaving the NNU, but this expertise and knowledge was not always recognised by other healthcare professionals who sometimes saw them as new and naïve parents. Enabling a clearer transition between neonatal and paediatric settings, which recognises the unique and vital experiences of these families and is underpinned by support and collaboration to share best practice between neonatal and paediatric teams, would bring clinical and experiential benefits for all concerned. For some families, leaving hospital was an anxious time and some felt lacking in confidence to care for their child without the safety net of the clinical environment, rather than because they did not know their child. Our findings build on the limited research which has been undertaken in families admitted to NNU and PICU which described the transition as a ‘culture clash’ [19] and supports the need for beginning preparation for NNU discharge and possible future PICU admissions from the start of the NNU admission [20].

### 4.3 The ‘Expert’ Parent

An alternative interpretation is that the limited incorporation of parental knowledge reflects legitimate concerns regarding clinical accountability and safety. In PICU, where children are acutely unwell, staff may be reluctant to share responsibility or rely on non‐professional knowledge. However, parents in this study described situations where their prior knowledge could have informed care decisions or prevented adverse events. This indicates that excluding parental expertise may itself carry risks, particularly for children with complex or multiple long‐term conditions.

Our findings also contribute to the concept of the ‘expert parent’. Existing literature often frames parents as becoming more knowledgeable over time and able to identify deterioration [21]. Our data suggest that expertise is not only developed but must also be recognised and legitimised within clinical encounters. Expertise is therefore relational and negotiated. In PICU, prior knowledge developed in NNU or at home was often not transferable unless validated by professionals. This has implications for safety, communication, and family‐centred care, as the value of parental knowledge depends on whether it is acknowledged and integrated into clinical decision‐making.

Contextual factors further shape these dynamics. The sample was largely homogeneous, and most participants were able to articulate concerns confidently. It is therefore likely that the tensions identified here may be amplified for families who face structural barriers, such as language differences or limited health literacy. This raises questions about equity in family‐centred care, and whose knowledge is heard and acted upon in clinical settings.

Taken together, these findings suggest that parental knowledge should not be viewed as supplementary to professional expertise, but as a distinct and valuable source of information that evolves across care pathways. Greater attention is needed to how this knowledge is recognised, validated and incorporated into care, particularly during transitions between services.

### 4.4 Strengths and Limitations

This study was conducted with attention to established criteria for trustworthiness in qualitative research, including credibility, transferability, dependability and confirmability [6]. Credibility was supported through in‐depth interviews, iterative data analysis and regular discussion within the research team to challenge interpretation. However, we did not undertake member checking and therefore cannot confirm that the findings fully reflect participants' intended meanings. Transferability is limited by the characteristics of the sample. Participants were primarily recruited via charities and were mostly White British and English‐speaking and this is often a challenge in paediatric research [22]. As a result, the findings may not reflect the experiences of more diverse populations, particularly those who may face barriers to participation or advocacy within healthcare settings. Dependability was enhanced through a transparent and systematic analytic process. However, interviews captured experiences that occurred up to 10 years previously. This introduces the possibility of recall bias and means that findings may reflect both historical and current healthcare practices. However, it is known that memories remain strong over time [23]. Confirmability may be influenced by the researchers' backgrounds. Although reflexive practices were embedded within the research process, the lead researcher's professional experience in paediatric research may have shaped data interpretation. Finally, this study reflects only parental perspectives. The absence of healthcare professional perspectives limits the ability to triangulate findings and fully understand how parental knowledge is interpreted within clinical decision‐making.

## 5 Implications for Further Research

Future research should focus on developing and evaluating interventions that support the systematic integration of parental expertise across care settings, particularly during transitions.

## 6 Conclusions

This study demonstrates that parental knowledge of their child develops dynamically across neonatal and paediatric intensive care and plays a central role in care and decision‐making. However, this knowledge is not consistently recognised across settings, particularly during transitions between services. The findings indicate that parental expertise is relational and context‐dependent, requiring active recognition by healthcare professionals to be effectively integrated into care. Where this recognition is absent, opportunities for communication, safety and continuity may be compromised. These findings are transferable to other high‐acuity paediatric contexts in which families provide ongoing and complex care. Embedding structured approaches to recognising and incorporating parental knowledge may strengthen family‐centred care and improve patient safety.

## Author Contributions

**Sarah E. Seaton:** conceptualisation, analysis, writing original draft and funding acquisition. **Ashleigh E. Butler:** validation and writing, reviewing and editing. **Joseph C. Manning:** validation and writing, reviewing and editing. **Lucy K. Smith:** validation and writing, reviewing and editing. **Katie Gallagher:** validation and writing, reviewing and editing. **Nicola Mackintosh:** validation and writing, reviewing and editing, and supervision.

## Funding

Sarah Seaton (Advanced Fellowship: NIHR300579) was funded by the National Institute for Health Research for this research project. The views expressed in this publication are those of the authors and not necessarily those of the NIHR, NHS or the UK Department of Health and Social Care.

## Ethics Statement

Ethics approval was granted by the University of Leicester Medicine and Biological Sciences Research Ethics Committee (reference: 32037‐ses26‐ls:healthsciences).

## Conflicts of Interest

The authors declare no conflicts of interest.

## Data Availability

Research data are not shared.

## Appendix 1 Summary of the Topic Guide

A summary of the questions covered in the topic guide is provided below:

- Please tell me about your child and your journey through pregnancy, birth and admissions to neonatal and paediatric intensive care
- Can you tell me a little about your child at the time they were admitted to PICU (e.g., age, how you came to realise they were unwell)?
- Can you describe the time you and your child spent on the PICU?
- After your time in the PICU, tell me about any additional hospital experience (e.g., time on a ward, ambulance transfer to home)?
- Can you tell me about the final day in hospital and how you prepared to go home from hospital?

What key messages do you have for other families going through the same experience, or healthcare professionals who care for them?

## References

[1] K. E. Best , N. Miller , E. Draper , D. Tucker , K. Luyt , and J. Rankin , “The Improved Prognosis of Hypoplastic Left Heart: A Population‐Based Register Study of 343 Cases in England and Wales,” Frontiers in Pediatrics 9 (2021): 635776. 34295856 10.3389/fped.2021.635776 PMC8289898

[2] S. Santhakumaran , Y. Statnikov , D. Gray , C. Battersby , D. Ashby , and N. Modi , “Survival of Very Preterm Infants Admitted to Neonatal Care in England 2008–2014: Time Trends and Regional Variation,” Archives of Disease in Childhood ‐ Fetal and Neonatal Edition 103 (2018): F208–F215. 28883097 10.1136/archdischild-2017-312748 PMC5916099

[3] C. Waddington , N. R. Veenendaal , K. O'Brien , and N. Patel , “Family Integrated Care: Supporting Parents as Primary Caregivers in the Neonatal Intensive Care Unit,” Pediatric Investigation 5 (2021): 148–154. 34179713 10.1002/ped4.12277 PMC8212757

[4] K. Meert , J. Clark , and S. Eggly , “Family‐Centered Care in the Pediatric Intensive Care Unit,” Pediatric Clinics of North America 3 (2013): 761–772. 10.1016/j.pcl.2013.02.011 PMC3767974 23639667

[5] K. Terp and A. Sjöström‐Strand , “Parents' Experiences and the Effect on the Family Two Years After Their Child Was Admitted to a PICU—An Interview Study,” Intensive & Critical Care Nursing 43 (2017): 143–148. 28663106 10.1016/j.iccn.2017.06.003

[6] Y. S. Lincoln and E. G. Guba , Naturalistic Inquiry (Sage Publ, 1985).

[7] M. M. Hennink , B. N. Kaiser , and V. C. Marconi , “Code Saturation Versus Meaning Saturation,” Qualitative Health Research 27 (2017): 591–608. 27670770 10.1177/1049732316665344 PMC9359070

[8] A. Tong , P. Sainsbury , and J. Craig , “Consolidated Criteria for Reporting Qualitative Research (COREQ): A 32‐Item Checklist for Interviews and Focus Groups,” International Journal for Quality in Health Care 19 (2007): 349–357. 17872937 10.1093/intqhc/mzm042

[9] D. F. Vears and L. Gillam , “Inductive Content Analysis: A Guide for Beginning Qualitative Researchers,” Focus on Health Professional Education 23 (2022): 111–127.

[10] C. Dressler , “The Psychological Implications of Having an Infant in the NICU for Mothers and Fathers,” Journal of Neonatal Nursing 30 (2024): 467–469.

[11] G. Colville and C. Pierce , “Patterns of Post‐Traumatic Stress Symptoms in Families After Paediatric Intensive Care,” Intensive Care Medicine 38 (2012): 1523–1531. 22706918 10.1007/s00134-012-2612-2

[12] G. Colville and C. M. Pierce , “Post‐Traumatic Stress Trajectories of Children and Their Parents Over the Year Following Intensive Care Discharge: A Secondary Analysis,” Nursing in Critical Care 29 (2023): 830–834. 37994217 10.1111/nicc.13014

[13] L. Dudek‐Shriber , “Parent Stress in the Neonatal Intensive Care Unit and the Influence of Parent and Infant Characteristics,” American Journal of Occupational Therapy 58 (2004): 509–520. 10.5014/ajot.58.5.509 15481778

[14] S. Saberian , C. Gale , N. Subhedar , et al., “Inequalities in Neonatal Unit Mortality in England and Wales Between 2012 and 2022: A Retrospective Cohort Study,” Lancet Child & Adolescent Health 9 (2025): 857–867. 41198365 10.1016/S2352-4642(25)00243-3

[15] H. K. Mitchell , S. E. Seaton , C. Leahy , et al., “Contribution of Ethnicity, Area Level Deprivation and Air Pollution to Paediatric Intensive Care Unit Admissions in the United Kingdom 2008–2021,” EClinicalMedicine 75 (2024): 102776. 39246717 10.1016/j.eclinm.2024.102776 PMC11377131

[16] R. I. Cook , M. Render , and D. D. Woods , “Gaps in the Continuity of Care and Progress on Patient Safety,” BMJ 320 (2000): 791–794. 10720370 10.1136/bmj.320.7237.791 PMC1117777

[17] T. Greenhalgh , R. Snow , S. Ryan , S. Rees , and H. Salisbury , “Six ‘Biases’ Against Patients and Carers in Evidence‐Based Medicine,” BMC Medicine 13 (2015): 200. 26324223 10.1186/s12916-015-0437-x PMC4556220

[18] J. K. O'Hara , K. Aase , and J. Waring , “Scaffolding Our Systems? Patients and Families ‘Reaching in’ as a Source of Healthcare Resilience,” BMJ Quality and Safety 28 (2019): 3–6. 10.1136/bmjqs-2018-008216 29764929

[19] R. Evans and B. Madsen , “Culture Clash: Transitioning From the Neonatal Intensive Care Unit to the Pediatric Intensive Care Unit,” Newborn and Infant Nursing Reviews 5 (2005): 188–193.

[20] S. P. Osorio Galeano and Á. M. Salazar Maya , “Preparing Parents for Discharge From the Neonatal Unit, the Transition, and Care of Their Preterm Children at Home,” Investigacion y Educacion en Enfermeria 41, no. 1 (2023): e04. 10.17533/udea.iee.v41n1e04 PMC10152913 37129352

[21] E. Mills , P. Lin , M. Asghari‐Jafarabadi , A. West , and S. Craig , “Association Between Caregiver Concern for Clinical Deterioration and Critical Illness in Children Presenting to Hospital: A Prospective Cohort Study,” Lancet Child & Adolescent Health 9 (2025): 450–458. 40451224 10.1016/S2352-4642(25)00098-7

[22] A. E. Butler , L. Ridgway , E. M. Henderson , et al., “Family‐Centred Care Research in Paediatrics: A Scoping Review,” Journal of Child Health Care 30 (2026): 150–166. 40298884 10.1177/13674935251337492 PMC12982550

[23] L. Provenzi and E. Santoro , “The Lived Experience of Fathers of Preterm Infants in the Neonatal Intensive Care Unit: A Systematic Review of Qualitative Studies,” Journal of Clinical Nursing 24 (2015): 1784–1794. 25850518 10.1111/jocn.12828

